# Photon-counting CT versus energy-integrating CT of the chest in acute respiratory distress syndrome: A retrospective observational study of image quality and radiation exposure

**DOI:** 10.1007/s10140-026-02490-2

**Published:** 2026-05-13

**Authors:** Daniel Rosok, Marcel Opitz, Denise Bos, Laura Klüner, Marcel Drews, Luca Salhöfer, Johannes Harmes, Nina Pirschtat, Simon Dubler, Michael Forsting, Johannes Haubold, Sebastian Zensen

**Affiliations:** 1 Institute of Diagnostic and Interventional Radiology and Neuroradiology, University Hospital Essen, University of Duisburg-Essen, Essen, Germany; 2 Department of Anesthesiology and Intensive Care Medicine, University Hospital Essen, University of Duisburg-Essen, Essen, Germany; 3 Institute of Diagnostic and Interventional Radiology, University Hospital Zurich, Zurich, Switzerland

**Keywords:** Lung injury, Photon-counting detectors, Chest computed tomography, Radiation dosage, Effective dose

## Abstract

**Purpose:**

Chest CT is central to the diagnosis and monitoring of acute respiratory distress syndrome (ARDS). Photon-counting detector CT (PCD-CT) may improve image quality and enable radiation dose optimization compared with Energy-integrating detector CT (EID-CT). However, evidence in critically ill ARDS patients undergoing contrast-enhanced chest imaging is limited. The purpose of this study was to intraindividually compare image quality and radiation exposure between PCD-CT and EID-CT in ARDS patients.

**Methods:**

This retrospective study included patients with ARDS who underwent both contrast-enhanced chest PCD-CT and EID-CT between April 2023 and May 2025. CT dose index volume (CTDIvol), dose–length product (DLP), effective and organ doses were extracted from dose monitoring software. Subjective image quality and diagnostic acceptability were rated independently by two radiologists using a Likert scale; inter-reader agreement was assessed with Cohen’s κ. Objective image quality was assessed using signal-to-noise ratios in representative anatomical regions. Intraindividual comparisons were performed using the Wilcoxon signed-rank test.

**Results:**

Among 531 ARDS patients, 80 (15.1%) underwent chest PCD-CT, including 59 (73.8%) contrast-enhanced examinations (median age: 51.7 years (IQR: 41.6–59.5); 62.7% male). Intraindividual imaging was performed in 32 patients (54.2%). PCD-CT demonstrated significantly higher subjective and objective image quality than EID-CT (*p* < 0.005), with moderate inter-reader agreement. Median CTDIvol and DLP were not significantly different between PCD-CT (8.2 mGy; 435 mGycm) and EID-CT (7.7 mGy; 297 mGycm).

**Conclusion:**

In contrast-enhanced chest CT of ARDS patients, PCD-CT provides superior image quality without a significant difference in radiation dose, potentially facilitating more precise diagnostic assessment in this critically ill population.

**Trial registration:**

DRKS-ID: DRKS00037916, 15 September 2025, retrospectively registered.

**Graphical abstract:**

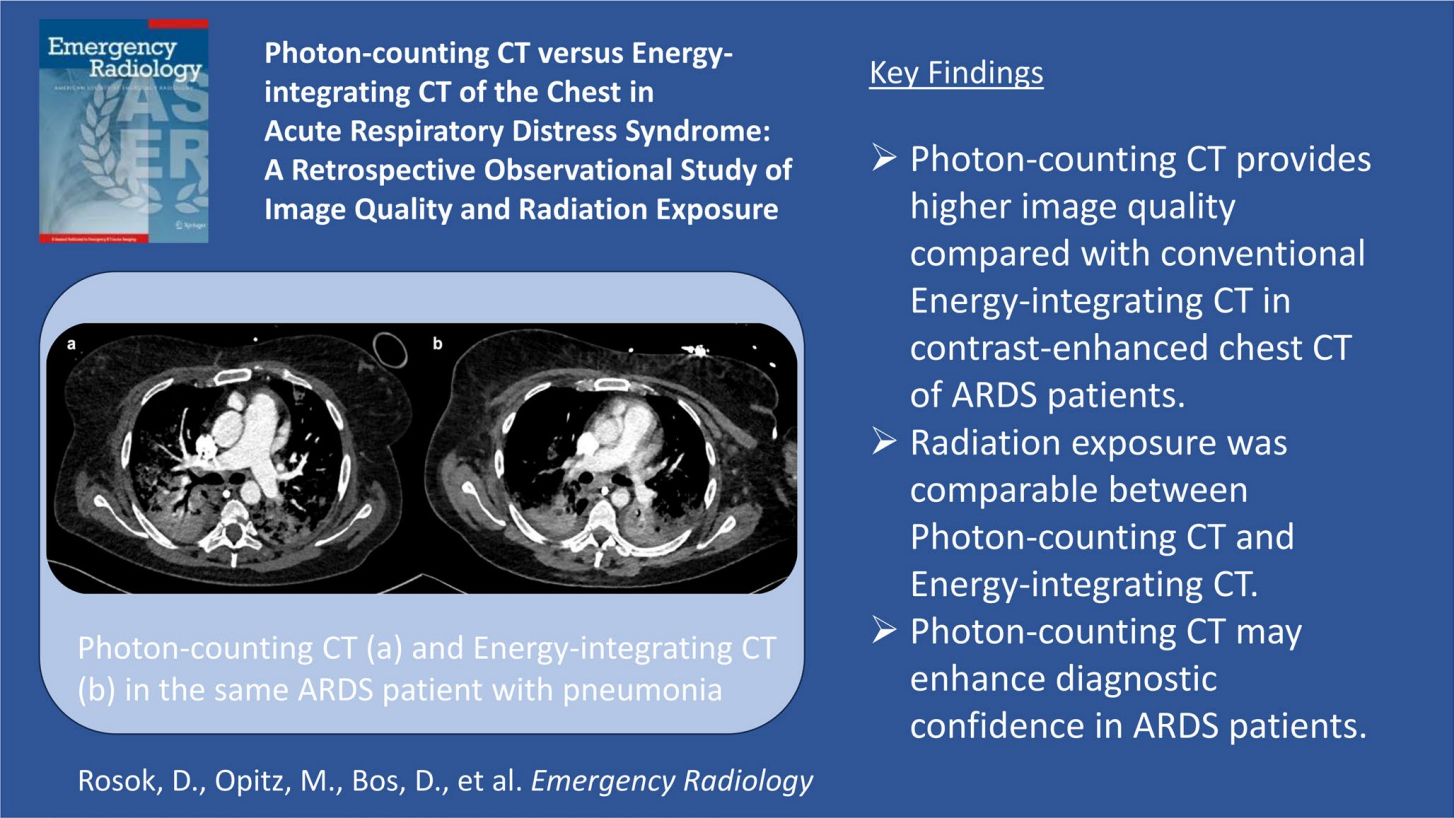

## Introduction

Acute respiratory distress syndrome (ARDS) is a life-threatening form of acute lung injury and carries a mortality rate of approximately 40% [1–6]. It is characterized by rapid-onset inflammatory pulmonary edema, severe hypoxemia, reduced lung compliance, and ventilation–perfusion mismatch [2, 7, 8]. ARDS is defined according to the Global definition, which incorporates oxygenation criteria to stratify severity into mild, moderate, and severe categories [6]. Accurate assessment of lung pathology is crucial for both diagnosis and management, and computed tomography (CT) provides the most detailed imaging evaluation.

Photon-counting detector (PCD) CT has been shown to improve image quality while maintaining adequate radiation dose efficiency [9, 10]. These characteristics make PCD-CT particularly appealing for patients with ARDS, who frequently undergo repeated chest CT examinations, in whom diagnostic image quality is a key consideration given the severity of disease. Precise CT imaging offers critical insights into lung morphology and aeration patterns in ARDS, which can inform ventilatory strategies and detect potential complications [8].

Several studies have characterized CT findings in ARDS and examined their associations with clinical outcomes [11–13]. Most prior studies have focused on non-contrast or low-dose CT protocols, and data on contrast-enhanced imaging—which is important for identifying complications such as pulmonary embolism—are limited [13, 14]. While Energy-integrating detector (EID) CT remains the clinical standard and PCD-CT has demonstrated advantages in chest imaging [15–18], evidence regarding its use specifically in patients with ARDS is still lacking. Moreover, detailed effective and organ-specific radiation dose data in ARDS patients remains scarce.

To address these gaps, the primary objective of this study was to compare image quality and radiation exposure between PCD-CT and conventional EID-CT in contrast-enhanced chest imaging of patients with ARDS. Secondary objectives included the evaluation of patient demographics, clinical characteristics, and CT imaging findings. By quantifying differences in these parameters, this study aims to provide a comparative assessment of image quality and radiation exposure between PCD-CT and EID-CT in critically ill patients with ARDS.

## Materials and methods

### Study design and patient population

The study was conducted according to the guidelines of the Declaration of Helsinki and approved by the local ethics committee (9 September, 2025; 25–12689-BO). Informed consent was waived due to the retrospective study design and all examinations being performed during routine clinical care. Patient data were anonymized. The study design was a retrospective, single-center, observational cohort study with consecutive patient selection. It was conducted according to the Strengthening the Reporting of Observational Studies in Epidemiology (STROBE) guidelines for observational studies [19, 20].

All patients between April 2023 and Mai 2025 with confirmed diagnosis of ARDS due to the Global criteria and a chest CT were screened [6]. Only patients that received a contrast-enhanced chest CT at the PCD-CT and an EID-CT during the same stay and a maximum interval of four weeks between the two examinations were included into the study. Patients were transferred from the Department of Anesthesiology and Intensive Care Medicine of our university hospital—a specialized supra-regional ARDS center—to our radiology department. CT scans of ARDS patients at our facility are performed immediately before and during intensive care unit (ICU) stay for diagnosis and therapy monitoring.

### Clinical data and diagnosis

Clinical data was extracted from the radiology and ICU reports. The following clinical data were collected: age, sex, presumed etiology of ARDS, presence of sepsis, use of ECMO therapy, and survival. The ARDS diagnosis was made by the ICU team based on the Global definition [6]. The cause of pneumonia was determined by using bacteriological data. Sepsis was diagnosed according to the latest Sepsis-3 definition [21].

### CT scanning protocols and image acquisition

In our institution, patients with suspected ARDS undergo contrast-enhanced chest CT in an arterial “mixed” phase, designed to provide simultaneous high contrast enhancement of the pulmonary arteries, aorta, and lung parenchyma. All CT examinations are acquired with the patient in the supine position. The indication for contrast-enhanced chest CT was assessment of the lung parenchyma and pulmonary perfusion, as well as exclusion of pulmonary arterial embolism.

#### Photon-counting detector CT

The scans were performed on a first-generation dual-source PCD-CT scanner with quantum imaging technology (NAEOTOM Alpha, Siemens Healthineers, Erlangen, Germany) covering the chest from the apex to the upper abdomen. An in-house standard scan protocol was applied with a tube voltage of 140 kVp. Other parameters were: automatic tube current modulation on (Care Dose4D, Siemens Healthineers, Erlangen, Germany) with image quality level (IQL) 80, 0.4 mm collimation, 0.25 s rotation time, and a pitch of 1.2. An iterative reconstruction algorithm was applied on level 3 (Quantum iterative reconstruction, Siemens Healthineers, Erlangen, Germany). Images were reconstructed with an axial slice thickness of 1 mm using a soft-tissue reconstruction kernel (Br40). Scans were initiated using automated bolus tracking in the descending aorta. Intravenous contrast medium (Ultravist 300, iopromide; Bayer Healthcare, Berlin, Germany) was administered according to patient weight at a flow rate of 4 ml/s, followed by a saline flush.

#### Energy-integrating detector CT

EID-CT scans were performed on three modern third-generation CT scanners by Siemens Healthineers, Erlangen, Germany (1: SOMATOM X.ceed, 2: SOMATOM Definition Edge, and 3: SOMATOM Definition AS+). All scans were performed using automated tube current modulation based on patient size, as determined from the anterior–posterior topogram (CARE Dose4D, Siemens Healthineers, Erlangen, Germany). Scan parameters of the three EID-CTs were as follows: tube voltage 1: 140 kVp, 2 and 3: 100 kVp; reference tube current: 1: 145 IQL, 2: 153 mAs, 3: 130 mAs; collimation: 1–3: 0.6; rotation time: 1: 0.25 s, 2: 0.28 s, 3: 0.3 s, and pitch: 1: 0.8, 2 and 3: 0.6. Data were reconstructed using an iterative reconstruction algorism on level 3 (ADMIRE, Siemens Healthineers, Erlangen, Germany). Images were reconstructed with an axial slice thickness of 1 mm using a soft-tissue reconstruction kernel (Br40). Contrast-enhanced scans were performed using the same standardized intravenous contrast protocol as described for PCD-CT, including bolus tracking in the descending aorta. Acquisition and reconstruction parameters were harmonized as closely as possible between both CT modalities to allow meaningful comparison.

### Image quality

Two radiologists with five years of experience in CT imaging of ARDS patients independently assessed the subjective image quality of contrast-enhanced chest CT scans from the same ARDS patients on both PCD-CT and EID-CT using a six-point Likert scale. Both readers had two and a half years of experience in the interpretation of PCD-CT examinations. Each examination was rated according to five parameters: (1) sharpness, (2) contrast, (3) noise, (4) artifacts, and (5) overall diagnostic acceptability, using the following scale: 6 = excellent, 5 = high, 4 = fully acceptable, 3 = mostly acceptable, 2 = suboptimal, 1 = unacceptable. For the rating of artifacts, the following scale was used: 6 = no or almost no artifacts, 5 = minimal artifacts, 4 = mild artifacts, 3 = moderate artifacts, 2 = pronounced artifacts, 1 = severe artifacts. All images were reviewed on the same Picture Archiving and Communication System (PACS) workstation (Centricity Universal Viewer 7, GE Healthcare, Chicago, IL, USA) with identical window settings (center: 50 Hounsfield units (HU), width: 350 HU) and field of view (approximately 400 × 400 mm) and on axial 1 mm soft tissue kernels (both Br40). Readers were fully blinded to scanner type, clinical information, and patient identity and evaluated all examinations independently in a randomized order.

For objective image quality analysis, signal-to-noise ratios (SNR) were calculated using regions of interest (ROIs) of comparable size (20–25 mm), positioned in corresponding anatomical locations across both modalities. ROIs were placed in predefined regions, including thoracic aorta, pulmonary trunk and lung parenchyma, while avoiding vessels, large bronchi, and imaging artifacts. Mean attenuation (HU) and standard deviation (SD) were recorded for each ROI. Image noise was defined as the SD of attenuation values, and SNR was calculated as the ratio of mean attenuation to image noise.

### Radiation dose

The CT dose index volume (CTDIvol) and dose-length product (DLP) were extracted from Digital Imaging and Communications in Medicine protocols archived in the PACS. Estimated effective doses (ED) and organ doses were obtained from the dose monitoring software Radimetrics Enterprise Platform (Bayer, Leverkusen, Germany), which is directly connected to the CT scanners and performs automated Monte Carlo–based dose simulations for all examinations. The platform selects the most appropriate standard reference phantom from a library of 18 Cristy-based models, using patient characteristics such as sex, age, and effective diameter to estimate organ doses [22]. The ED was calculated by Radimetrics using tissue-weighting factors defined in International Commission on Radiological Protection Publication 103, applied to the simulated organ doses [23, 24].

### Statistical analysis

Data were analyzed using Statistical Package for Social Sciences Version 29.0 (SPSS, IBM Corp, Armonk, New York, USA; RRID: SCR_016479). Continuous variables were assessed for normality using the Shapiro–Wilk test and are reported as mean ± SD or median (IQR), as appropriate. Categorical variables are presented as counts and percentages. Intra-individual comparisons of radiation dose parameters and image quality between PCD-CT and EID-CT were performed using the Wilcoxon signed-rank test. Inter-reader agreement for image quality was evaluated with Cohen κ. Two-sided p-values < 0.05 were considered statistically significant. Figures were created using GraphPad Prism Version 5.0 (GraphPad Software, La Jolla, California, USA; RRID: SCR_002798).

## Results

### Study population

531 ARDS patients received a chest CT between April 2023 and May 2025 and 15.1% (80/531) underwent PCD-CT. Of these, 73.8% (59/80) patients received contrast-enhanced examinations (median age: 51.7 years (IQR: 41.6–59.5, range: 23.2–79.5); 62.7% (37/59) male). In 54.2% (32/59), contrast-enhanced scans were available for both PCD-CT and EID-CT. The median interval between PCD-CT and EID-CT examinations was 9 days (range 1–28 days).

### Clinical data and diagnosis

Severe ARDS, according to the Global definition, was present in 88.1% (52/59), while 11.9% (7/59) had moderate ARDS; none of the patients met criteria for mild ARDS. All patients were diagnosed with pneumonia at admission or during the course of hospitalization. Across the cohort, 21 different primary pathogens were identified, and mixed infections with multiple organisms were common. A bacterial pathogen was identified in 55.9% (33/59) of patients, a viral pathogen in 33.9% (20/59), while no pathogen was identified in 10.2% (6/59). The distribution of identified pathogens is summarized in Table 1.

**Table 1:** Microbiological spectrum of pneumonia pathogens among ARDS patients

Pathogen		*n*
Bacterial		**55.9% (33/59)**
	Pseudomonas aeruginosa Klebsiella pneumoniae Streptococcus pneumoniae Legionella pneumophilia Staphylococcus aureus Enterococcus faecium Escherichia coli	6.8% (4/59) 6.8% (4/59) 5.1% (3/59) 5.1% (3/59) 5.1% (3/59) 5.1% (3/59) 3.4% (2/59)
	Mycoplasma pneumoniae Mycobacterium tuberculosis Streptococcus pyogenes Haemophilus influenzae Serratia marcescens Staphylococcus haemolyticus Staphylococcus hominis Stenotrophomonas maltophilia	3.4% (2/59) 3.4% (2/59) 1.7% (1/59) 1.7% (1/59) 1.7% (1/59) 1.7% (1/59) 1.7% (1/59) 1.7% (1/59)
	Gram-negative rods unspecified	1.7% (1/59)
Viral		**33.9% (20/59)**
	Influenza virus Cytomegalovirus COVID-19 Herpes simplex virus Adenoviruses	15.3% (9/59) 5.1% (3/59) 5.1% (3/59) 5.1% (3/59) 3.4% (2/59)
No pathogen identified		**10.2% (6/59)**

A concomitant sepsis was present in 88.1% (52/59) of patients, and 74.6% (44/59) underwent extracorporeal membrane oxygenation (ECMO) therapy. Of those receiving ECMO therapy, 95.5% (42/44) were treated with veno-venous ECMO and 4.5% (2/44) with veno-arterial ECMO. Overall cohort mortality was 67.8% (40/59). In patients with severe ARDS, mortality was 69.2% (36/52), with a median survival of 16 days (IQR: 9–33). In moderate ARDS, mortality was 57.1% (4/7), and the median survival time was 24 days (IQR: 11–106).

Cardiopulmonary resuscitation was required in 11.9% (7/59) of patients. Pneumothorax occurred in 25.4% (15/59), and pulmonary embolism in 18.6% (11/59). Among the pulmonary emboli, 90.9% (10/11) were peripheral. One patient (1.7%, 1/59) had a central pulmonary embolism accompanied by a lung abscess. No pulmonary emboli were identified on PCD-CT that had not been detected on EID-CT. Pleural empyema was identified in 3.4% (2/59), while bronchopulmonary hemorrhage and hemothorax occurred in 10.2% (6/59).

### Image quality

#### Subjective analysis

In 32 patients, subjective image quality assessment using a Likert scale demonstrated significantly higher scores for PCD-CT compared with EID-CT for sharpness, contrast, and overall diagnostic acceptability (all *p* < 0.001), as well as for noise (*p* = 0.005), whereas no statistically significant difference was observed for artifacts (*p* = 0.45). Interreader agreement was moderate (Cohen κ = 0.40). For PCD-CT, median Likert scores were 6 for sharpness, contrast, and overall diagnostic acceptability and 5 for noise and artifacts (all with ranges: 4–6). For EID-CT, median scores were 5 for sharpness, contrast, and diagnostic acceptability (all with ranges: 4–6), 4 for noise (ranges 3–5), and 5 for artifacts (ranges: 3–6). The largest differences in image quality ratings were observed for sharpness, contrast, and overall diagnostic acceptability, which demonstrated the largest effect sizes (*r* = 0.87) and modestly higher interreader agreement (Cohen κ = 0.50).

#### Objective analysis

In 32 patients, objective image quality assessment demonstrated significantly higher signal and SNR values for PCD-CT compared with EID-CT across all evaluated regions.

In the thoracic aorta, median signal was higher for PCD-CT (338.5 HU [IQR: 302.5–388.25]) than for EID-CT (218 HU [IQR: 183.5–267]; *p* < 0.001), while noise did not differ significantly (23 [IQR: 18.75–28.75] vs. 20 [IQR: 14–32.75]; *p* = 0.299). Consequently, SNR was significantly higher for PCD-CT (15.17 [IQR: 10.70–18.31]) compared with EID-CT (11.28 [IQR: 8.42–13.90]; *p* < 0.001).

In the pulmonary trunk, signal was also significantly higher for PCD-CT (374.5 HU [IQR: 302.25–454.75]) than for EID-CT (243 HU [IQR: 205–329.5]; *p* < 0.001), whereas noise showed no statistically significant difference (22 [IQR: 19–25.75] vs. 17 [IQR: 14–27.75]; *p* = 0.074). Correspondingly, SNR was significantly higher for PCD-CT (17.9 [IQR: 13.03–21.41]) compared with EID-CT (13.42 [IQR: 10.23–17.01]; *p* = 0.008).

**Fig. 1:**
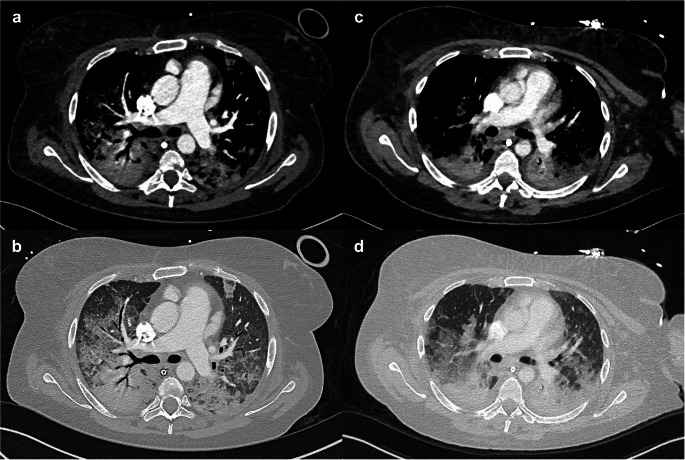
Contrast-enhanced chest Photon-counting detector CT (**a**, **b**) and Energy-integrating detector CT (**c**, **d**) obtained 7 days apart in the same female patient in her 40 s with ARDS and pneumonia

In the lung parenchyma, PCD-CT again demonstrated higher signal (84.5 HU [IQR: 60.75–143.5] vs. 56 HU [IQR: 39.25–70]; *p* < 0.001), with no significant difference in noise (27 [IQR: 20–37.75] vs. 22.5 [IQR: 16.75–35.5]; *p* = 0.115). SNR was significantly higher for PCD-CT (3.45 [IQR: 2.66–4.39]) than for EID-CT (2.57 [IQR: 1.69–3.20]; *p* < 0.001). Figure 1 illustrates the superior image quality of PCD-CT compared to EID-CT, with notably increased contrast and spatial sharpness.

### Radiation dose

In 59 patients with ARDS who underwent contrast-enhanced chest PCD-CT, median CTDIvol was 9.3 mGy (IQR: 5.8–12.6) and median DLP was 427 mGycm (IQR: 234–666) (Table 2). Most patients (86.4%, 51/59) underwent more than one CT examination during the same hospitalization, with a median of 3 chest CT scans per patient (IQR: 1–3). No patient was duplicated within the cohort.

In 32 patients with an additional contrast-enhanced chest CT performed on an EID-CT, scanner-specific analyses were performed (Table 2). Intraindividual radiation doses did not differ significantly between PCD-CT and the pooled median of three EID-CTs, with slightly higher median values observed for PCD-CT: CTDIvol 8.2 mGy (IQR: 6.3–12.6) vs. 7.7 mGy (IQR: 5.4–11.3; +15.2%, IQR: − 38.1–31.2; *p* = 0.62) and DLP 435 mGycm (IQR: 254–603) vs. 297 mGycm (IQR: 218–631; +23.5%, IQR: − 38.2–49.2; *p* = 0.36).

**Table 2:** Radiation dose parameters of contrast-enhanced chest CT in ARDS patients among different scanners

CT scanner	Radiation dose parameter		
NAEOTOM Alpha		n	59
	CTDIvol^a^ (mGy)	Median	9.3
		IQR^c^	5.8–12.6
	DLP^b^ (mGycm)	Median	427
		IQR	234–666
SOMATOM X.ceed		n	7
	CTDIvol (mGy)	Median	5.6
		IQR	4.1–7.5
	DLP (mGycm)	Median	264
		IQR	212–511
SOMATOM Definition Edge		n	18
	CTDIvol (mGy)	Median	10.8
		IQR	7.5–16.5
	DLP (mGycm)	Median	366
		IQR	252–720
SOMATOM Definition AS+		n	7
	CTDIvol (mGy)	Median	6.3
		IQR	4.9–8.9
	DLP (mGycm)	Median	186
		IQR	159–631

^a^CT dose index volume referenced to a 32 cm CTDI phantom, ^b^dose-length product, ^c^interquartile range

**Fig. 2:**
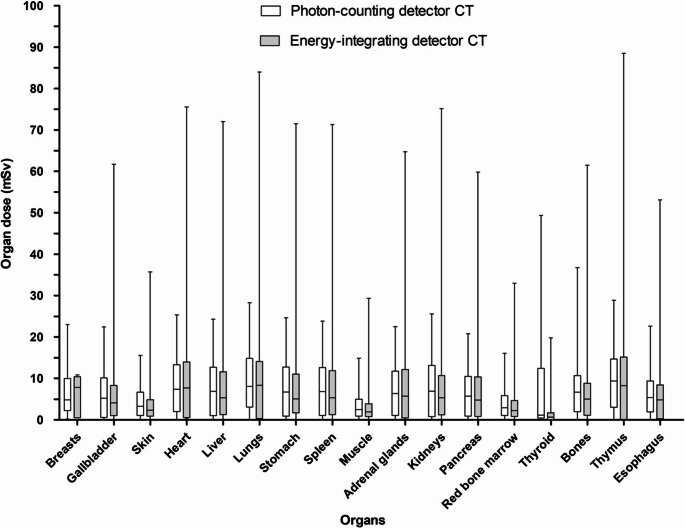
Intraindividual Comparison of Chest CT Organ doses in ARDS patients. Whiskers indicate minimum and maximum values

Organ dose estimates were available via the dose monitoring software for 66.1% (39/59) of PCD-CT examinations and 40.7% (24/59) of EID-CT examinations (Table 3). In 17 of 59 patients (32.7%), both ED and organ dose estimates from irradiated organs were available from Radimetrics for PCD-CT and EID-CT, allowing intraindividual dose comparison (Table 4). In this paired cohort, neither ED nor organ doses differed significantly between PCD-CT and EID-CT using the applied protocols (Table 4; Fig. 2). Median ED was 4.09 mSv (IQR: 1.43–7.10) for PCD-CT and 3.81 mSv (IQR: 0.92–6.15) for EID-CT (*p* = 0.62).

**Table 3:** Organ Doses of Chest CT in ARDS Patients

Organs	Photon-counting detector CT (*n* = 39)		Energy-integrating detector CT (*n* = 24)	
	Median (mSv)	Interquartile range	Median (mSv)	Interquartile range
Thyroid	1.34	0.73–4.66	0.51	0.01–1.40
Breasts	8.49	4.83–16.58	5.23	0.52–9.91
Heart	8.96	4.02–13.87	6.71	0.46–12.12
Lungs	9.65	4.63–15.50	6.55	0.31–11.46
Esophagus	6.20	3.31–9.42	3.81	0.23–6.68
Adrenal glands	7.77	3.58–12.10	5.30	0.18–10.01
Kidneys	7.59	2.96–12.73	5.12	0.04–8.04
Liver	8.01	3.33–12.52	5.11	0.15–9.08
Gall bladder	5.95	2.14–9.58	3.86	0.05–5.86
Spleen	8.09	3.49–12.93	5.13	0.12–9.59
Pancreas	6.89	2.91–10.88	4.38	0.13–8.46
Stomach	7.66	3.14–12.71	4.44	0.10–8.22
Small intestine	1.08	0.37–2.38	0.50	0.01–2.45
Colon	0.98	0.34–2.17	0.47	0.01–2.22
Skin	3.30	1.86–6.32	2.20	0.10–4.11
Bones	6.48	4.06–10.54	4.28	0.21–7.14
Red bone marrow	3.31	2.01–5.67	2.04	0.11–3.59
Muscles	2.85	1.73–4.78	1.89	0.07–2.98

**Table 4:** Intra-individual Comparison of Organ Doses from Chest CT in ARDS Patients

Organs	Photon-counting detector CT (*n* = 17)		Energy-integrating detector CT (*n* = 17)		Photon-counting detector CT vs. Energy-integrating detector CT (*n* = 17)		
	Median (mGy)	Interquartile range	Median (mGy)	Inter- quartile range	Percentage difference	Interquartile range	*p*-value
Thyroid	0.06	0.02–0.42	0.68	0.01–1.71	48.2	−995.7–98.6	0.29
Breasts	4.83	2.24–9.99	7.83	0.52–10.38	0.5	−1680.5–79.8	0.60
Heart	7.4	2.01–13.28	7.71	0.55–13.95	7.6	−636.0–88.0	0.44
Lungs	8.08	3.10–14.86	8.38	0.35–14.06	6.8	−637.8–91.6	0.52
Esophagus	5.38	1.94–9.40	4.83	0.25–8.44	26.6	−5910.1–94.0	0.65
Adrenal glands	6.36	1.08–11.76	5.75	0.46–12.15	16.2	−1168.3–95.4	0.44
Kidneys	6.92	0.81–13.16	5.34	1.23–10.69	31.8	−1681.3–80.4	0.62
Liver	6.86	1.06–12.68	5.30	1.29–11.59	22.2	−1230.7–88.7	0.44
Gall bladder	5.20	0.60–10.17	4.13	1.06–8.30	38.8	−2231.5–79.7	0.65
Spleen	6.83	1.07–12.61	5.31	1.26–11.89	17.7	−1450.2–89.1	0.44
Pancreas	5.74	0.95–10.52	4.79	0.83–10.37	21.8	−1180.6–90.9	0.44
Stomach	6.72	0.93–12.73	5.08	1.71–11.03	29.6	−1389.5–85.8	0.49
Small intestine	1.29	0.11–4.00	0.60	0.13–2.24	31.3	−1483.3–93.3	0.72
Colon	1.14	0.09–3.56	0.56	0.12–2.02	31.7	−1720.1–92.9	0.72
Skin	3.30	1.09–6.65	2.35	0.90–4.85	23.9	−626.0–85.5	0.80
Bones	6.66	1.98–10.68	5.03	1.11–8.83	23.1	−671.8–90.1	0.72
Red bone marrow	2.92	1.08–5.85	2.25	0.84–4.70	28.3	−668.8–88.0	0.72
Muscles	2.48	0.90–4.95	1.92	0.80–3.85	25.7	−699.6–85.4	0.83

## Discussion

This study demonstrates that PCD-CT provides superior image quality and diagnostic acceptability compared with EID-CT in patients with ARDS undergoing contrast-enhanced chest imaging, while radiation dose levels did not differ significantly. These findings are particularly relevant given the high clinical acuity, frequent complications, and intensive monitoring required in this population, where CT imaging can lead to changes in management in up to 26.5% of cases [13].

PCD-CT achieved significantly higher ratings for image sharpness, contrast, noise, and overall diagnostic acceptability, whereas the prevalence of image artifacts—largely due to medical devices in ARDS patients—was comparable between modalities. These improvements indicate that detector technology, rather than device-related confounders, accounted for enhanced image quality. This is further supported by the objective image quality analysis, which demonstrated significantly higher SNR values for PCD-CT across all evaluated regions, including the thoracic aorta, pulmonary trunk, and lung parenchyma, primarily driven by increased signal intensity while noise remained comparable between modalities. Consistent with our findings, Van Ballaer et al. demonstrated superior visualization of nearly all lung structures using PCD-CT compared with EID-CT [25].

Beyond image quality, the potential impact on lesion detection, particularly pulmonary embolism, requires cautious interpretation. In our cohort, EID-CT examinations demonstrated a high level of diagnostic acceptability, and no pulmonary emboli were identified on PCD-CT that had not been detected on EID-CT. However, a direct comparison of embolus detection between modalities is limited by the study design, as examinations were not performed on the same day but with a median interval of 9 days. During this period, initiation or continuation of anticoagulation therapy—especially in patients receiving ECMO—may have influenced the presence and appearance of emboli. Future prospective studies with same-day acquisitions, as performed by Prayer et al., may help to better delineate potential differences in lesion detection between modalities [18].

Radiation dose metrics did not differ significantly between PCD-CT and EID-CT. This supports the noninferiority of PCD-CT and provides a reliable basis for image quality assessment in this study. DLP values were numerically higher for PCD-CT. However, the differences were small and not statistically significant, suggesting that they are unlikely to translate into a clinically relevant impact on patient radiation exposure. Although the physical properties of PCD-CT allow potential dose reduction, maintaining diagnostic reliability in critically ill ARDS patients remains paramount [9]. Mohammadzadeh et al. demonstrated that PCD-CT can achieve higher image quality and lower radiation exposure compared with EID-CT [15], although intraindividual comparisons were not performed. In the present study focusing on contrast-enhanced CT in ARDS patients, often with extensive supportive devices such as ECMO, we observed a different pattern. This may be explained by the increased complexity of imaging in this specific clinical setting. Contrast enhancement leads to higher attenuation values, which can increase image noise and trigger higher tube current in automated dose modulation. In addition, ARDS-related alterations of lung parenchyma, including consolidation and edema, result in higher and more heterogeneous attenuation, further affecting noise characteristics. The presence of medical devices such as ECMO circuits, catheters, and monitoring equipment may introduce additional artifacts and beam hardening effects. Furthermore, motion-related factors in critically ill and mechanically ventilated patients may necessitate more robust acquisition settings to ensure diagnostic image quality. Finally, differences in tube voltage and the current stage of protocol optimization for PCD-CT may also contribute to the observed dose pattern. Together, these findings indicate that radiation dose in PCD-CT is influenced by multiple interacting factors and that its potential dose advantage is context-dependent, representing an important consideration for clinical implementation and further optimization. Analogous to our study, Prayer et al. observed higher CTDIvol and DLP in PCD-CT compared with EID-CT in their intraindividual study on post-COVID lung abnormalities [18], likely reflecting differences in patient populations and technical settings.

Our cohort exhibited high clinical acuity, with 88.1% of patients presenting with severe ARDS. Pneumonia was present in all patients, bacterial pathogens were identified in over half of the cohort, and sepsis was common. While most ARDS patients exhibit diffuse alveolar damage, other histological patterns—including organizing pneumonia—may also occur [26]. Despite intensive therapy, including ECMO therapy in 74.6% of patients, mortality in our study remained high at 67.8%, comparable to reports in COVID-ARDS cohorts [27] and higher than rates in the LUNG SAFE study [4]. These findings underscore the importance of reliable imaging not only for parenchymal assessment but also for detecting clinically relevant complications such as pulmonary embolism. Contrast-enhanced CT is particularly valuable for this purpose, distinguishing our study from prior investigations focusing on non-contrast or low-dose protocols [18].

Conventional EID-CT relies on scintillators and photodiodes, whereas PCD-CT directly converts X-ray photons in a continuous semiconductor layer, enabling ultra-high-resolution imaging and spectral differentiation [9]. These capabilities enhance parenchymal evaluation, supporting ventilatory optimization, assessment of lung recruitability, and therapy monitoring. Although our study did not assess whether improved image quality led to additional clinically actionable findings, prior work shows that PCD-CT can detect subtle abnormalities missed on EID-CT [18].

Several studies suggest that CT findings may predict severity and mortality in ARDS [28–30]. Acute phase findings, including ground-glass opacities and consolidation, and fibrotic changes, such as traction bronchiectasis and honeycombing, have been associated with worse outcomes [28–30]. Semi-quantitative CT scores stratifying lobe involvement and infiltrate extent have been proposed to assess ARDS severity [31]. However, definitions of CT findings are not standardized, progression is heterogeneous, and therapeutic interventions—including fluid management, ventilation settings, or prone positioning—can influence imaging appearance. Notably, a recent meta-analysis concluded that CT findings alone may not reliably predict short-term mortality [11].

Clinical implications of this study include the potential for workflow optimization and scanner allocation. In centers with multiple CT systems available, preferential use of PCD-CT in ARDS patients may enhance diagnostic confidence and support individualized therapeutic decision-making, particularly in the context of this life-threatening disease that is still frequently underrecognized in clinical practice [4].

This study has limitations. The retrospective design, single-center setting, and relatively small sample size may limit generalizability. The small intraindividual sample size is primarily due to the gradual clinical implementation of PCD-CT at our institution, during which only a subset of patients underwent both PCD-CT and EID-CT examinations. This may introduce selection bias, as patients included in the intraindividual analysis were not randomly selected but reflect the availability of both imaging modalities. However, the intraindividual design enabled direct comparison between imaging modalities while minimizing interpatient variability. Dose estimates were available for a subset. The interval between PCD-CT and EID-CT varied by up to four weeks (median 9 days), which may have introduced confounding from changes in disease status and therapeutic interventions (e.g., ventilation or fluid management). Although acquisition and reconstruction parameters were harmonized as closely as possible between both CT modalities, minor differences in technical parameters cannot be entirely excluded and may have had an impact on direct comparability. Despite these limitations, this is, to our knowledge, the first study evaluating PCD-CT in ARDS patients, providing novel data on image quality, and detailed intraindividual effective and organ-specific radiation doses.

## Conclusion

PCD-CT enables higher image quality and diagnostic reliability for contrast-enhanced chest imaging in ARDS patients while maintaining radiation dose levels without a significant difference compared to conventional CT. In this critically ill population, these advantages may support more accurate and timely diagnostic assessment under challenging clinical conditions. Further studies are warranted to investigate whether the improved image quality influences clinical management decisions and whether such changes translate into measurable improvements in patient outcomes.

## Data Availability

The data are not openly available due to reasons of sensitivity and are available from the corresponding author upon reasonable request.
